# Bioenergetics-Driven Extracellular Vesicle Therapies for Heart Failure: From Preclinical Insights to Regenerative Translation

**DOI:** 10.3390/ijms27135849

**Published:** 2026-06-29

**Authors:** Dhienda C. Shahannaz, Tadahisa Sugiura

**Affiliations:** 1 Digestive Disease & Surgery Institute, Cleveland Clinic, Cleveland, OH 44195, USA; dhiendaladdynasrul@gmail.com; 2 Department of Cardiothoracic and Vascular Surgery, Montefiore Medical Center, Albert Einstein College of Medicine, New York City, NY 10467, USA

**Keywords:** heart failure, extracellular vesicles, cardiac bioenergetics, energetic insufficiency, metabolic remodeling, mitochondrial efficiency, systems cardiology, translational therapeutics, intercellular energy signaling, organ-level metabolism

## Abstract

Heart failure (HF) is fundamentally a disease of energetic insufficiency, in which impaired mitochondrial efficiency, maladaptive metabolic remodeling, and disrupted intercellular signaling converge at the organ level to limit cardiac performance. Despite advances in pharmacologic and device-based therapies, current treatment paradigms largely modulate hemodynamics or neurohormonal pathways rather than directly restoring myocardial bioenergetic capacity. Emerging evidence positions extracellular vesicles (EVs) as endogenous regulators of cardiac energy homeostasis, capable of orchestrating coordinated metabolic and mitochondrial adaptations across cardiac and non-cardiac cell populations. This review advances a system-level framework in which EVs are conceptualized as bioenergetic therapeutics, i.e., active biological agents that reprogram cellular energy utilization, substrate flexibility, and mitochondrial efficiency, rather than passive carriers of isolated molecular cargo. We synthesize preclinical evidence demonstrating EV-mediated modulation of oxidative phosphorylation, glycolytic balance, redox signaling, and mitochondrial dynamics, and examine how these effects scale from cellular and small-animal models to clinically relevant heart failure phenotypes. Importantly, we highlight organ-level integration, wherein EV signaling interfaces with vascular, immune, and metabolic networks to reshape myocardial energetic demand and supply. By bridging mechanistic insights with translational considerations, this review addresses the central question of how EV-driven bioenergetic reprogramming can be deployed within contemporary HF treatment paradigms. We propose EV-based strategies as complementary or synergistic interventions capable of restoring energetic resilience, reframing heart failure therapy beyond structural repair toward systemic metabolic renewal.

## 1. Introduction

### 1.1. Heart Failure as a Bioenergetic Disease

Heart failure represents the final common pathway of diverse cardiovascular insults, yet its progression is unified by a shared biological denominator: chronic energetic failure of the myocardium [1,2,3,4]. Long before overt systolic or diastolic dysfunction manifests, failing hearts exhibit impaired mitochondrial efficiency, reduced ATP availability, altered substrate utilization, and disrupted metabolic signaling across cardiomyocytes, endothelial cells, fibroblasts, and immune populations [5]. Heart failure is characterized by progressive energetic insufficiency, reflected by reductions in myocardial ATP availability and phosphocreatine reserves that correlate with disease severity and adverse clinical outcomes [3,4,6,7,8]. This energetic mismatch—between myocardial demand and bioenergetic capacity—limits contractile reserve, accelerates maladaptive remodeling, and constrains the efficacy of conventional therapies [3,4,5,9,10,11,12].

Over the past two decades, heart failure research has increasingly recognized metabolic remodeling as a driver rather than a bystander of disease progression [5,12,13,14,15]. Healthy adult myocardium relies predominantly on oxidative metabolism, whereas failing hearts undergo maladaptive substrate remodeling characterized by reduced metabolic flexibility and impaired mitochondrial energy production [1,2,3,5,9,10,12]. Simultaneously, mitochondrial oxidative phosphorylation becomes inefficient due to impaired electron transport chain (ETC) complex I and III activity [4], increased proton leak [4], and excessive reactive oxygen species (ROS) generation [3,4]. Mitochondrial fragmentation, driven by dysregulated DRP1 activation and reduced MFN1/2 and OPA1 expression [3,4,9,10,11,12], further compromises ATP production and calcium handling [3,4]. Collectively, these alterations reduce mitochondrial ATP yield per oxygen molecule by up to 25–30%, producing an energetically wasteful myocardium [16,17,18,19,20].

Despite these mechanistic insights, therapeutic translation has lagged behind. Existing pharmacologic approaches—such as β-blockers, ACE inhibitors, ARNI, and SGLT2 inhibitors [21,22,23]—improve outcomes partly through indirect metabolic effects, yet they do not directly restore myocardial bioenergetic capacity [24,25,26,27,28,29,30]. Mineralocorticoid receptor antagonists (MRAs) and diuretics likewise remain integral components of guideline-directed medical therapy, reducing adverse remodeling and congestion, respectively, although their effects on myocardial bioenergetic restoration are largely indirect. Trials targeting metabolism more directly, including fatty acid oxidation inhibitors or glucose modulators, have yielded modest or inconsistent benefits, often limited by systemic side effects [3,4,9,10,11,12]. A central limitation has been the absence of strategies capable of coordinating metabolic adaptation across cell types [4] and spatial scales within the heart, particularly in the context of chronic inflammation, fibrosis, and vascular dysfunction [3,4,31,32,33].

Extracellular vesicles (EVs) have emerged as critical mediators of intercellular communication in cardiovascular physiology and disease [34,35]. Traditionally framed as carriers of proteins, RNAs, and lipids, EVs are increasingly recognized for their ability to encode functional metabolic states and transmit them between cells [34,35,36]. Cardiac- and stem cell-derived EVs contain bioenergetically relevant cargo, including microRNAs (e.g., miR-21, miR-126, miR-210) [4,36], metabolic enzymes, mitochondrial regulatory proteins, and lipid species that modulate membrane composition and redox balance [5,34]. In heart failure models, EVs have been shown to enhance mitochondrial membrane potential, normalize NAD^+^/NADH ratios, increase citrate synthase activity, and improve oxygen consumption rates by 20–50% in recipient cardiomyocytes [37,38,39,40]. Importantly, these effects arise not from isolated molecular actions, but from integrated bioenergetic signaling programs that reshape cellular metabolism and stress responses.

This manuscript proposes a systems-biology perspective in which extracellular vesicles (EVs) function as bioenergetic regulators capable of coordinating metabolic adaptations across multiple cardiac cell populations [14]. Although current evidence remains largely preclinical, studies have demonstrated EV-mediated effects on mitochondrial function, angiogenic signaling, inflammatory responses, and fibrotic remodeling [34,35,36]. These interconnected mechanisms raise the possibility that EVs may influence myocardial energy balance not only through direct effects on cardiomyocytes but also through modulation of endothelial, immune, and stromal cell metabolism. Accordingly, we examine EVs as candidate bioenergetic therapeutics and discuss the translational implications of this emerging framework for heart failure management. This system-level perspective is essential for understanding how EV-mediated effects observed in preclinical models—often demonstrating improvements in ejection fraction of 5–15% and reductions in fibrotic burden of 20–40% [4]—may inform future therapeutic strategies for chronic heart failure.

**Figure 1:**
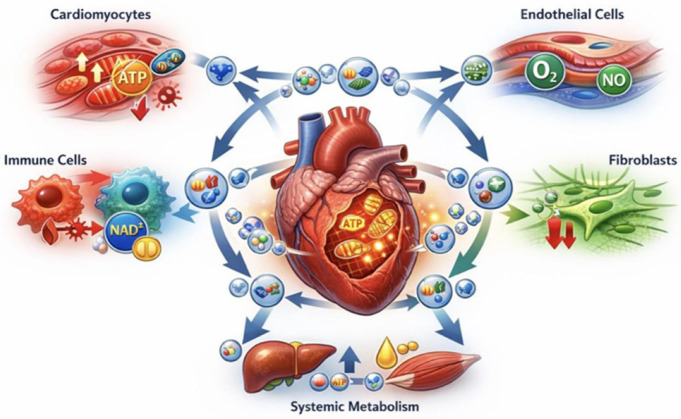
System-Level EV-Mediated Bioenergetic Reprogramming in Heart Failure. The schematic depicts extracellular vesicles (EVs) as central mediators of bioenergetic communication across the failing cardiac ecosystem. EV cargo, including microRNAs, proteins, lipids, and mitochondrial regulatory factors, modulates mitochondrial respiration, ATP production, redox homeostasis, and mitochondrial quality control within recipient cardiomyocytes. Simultaneously, EV-mediated signaling influences endothelial cells by enhancing angiometabolic coupling and substrate delivery, promotes reparative immune cell polarization that reduces inflammatory metabolic stress, and suppresses fibroblast activation associated with energetically unfavorable remodeling. Through these coordinated multicellular interactions, EVs improve myocardial energetic efficiency and metabolic resilience at the tissue and organ level. The figure illustrates the central concept that therapeutic EVs act as system-wide bioenergetic regulators rather than single-target molecular interventions.

Figure 1 presents the central conceptual framework of this review, illustrating how extracellular vesicles (EVs) coordinate bioenergetic regulation across multiple cardiac and extracardiac compartments. At the mitochondrial level, EV cargo influences oxidative phosphorylation, mitochondrial dynamics, redox balance, and ATP generation. Within cardiomyocytes, these effects support contractile efficiency and metabolic flexibility. Beyond cardiomyocytes, EV-mediated signaling modulates endothelial function, promotes angiometabolic coupling, attenuates fibroblast activation, and reprograms immune cell metabolism toward reparative phenotypes. These multicellular interactions collectively improve substrate delivery, reduce inflammatory metabolic burden, and enhance myocardial energetic reserve. The figure emphasizes that EVs function not as isolated molecular carriers but as system-wide coordinators of bioenergetic homeostasis, linking cellular metabolism to organ-level cardiac performance and providing the conceptual basis for their therapeutic application in heart failure.

Importantly, this review is intentionally distinct from regenerative strategies centered on infarct repair or whole-mitochondria delivery. While EV-based regenerative approaches for myocardial infarction emphasize structural restoration and cell replacement, heart failure demands interventions that restore energetic coordination and metabolic resilience across a remodeled and heterogeneous myocardium [1,13]. Here, we focus on how EV-driven bioenergetic reprogramming interfaces with vascular function, immune modulation, and systemic metabolism to support sustained cardiac performance rather than focal tissue repair.

By integrating preclinical evidence with translational frameworks, this paper addresses a critical gap in heart failure therapeutics: how to move from mechanistic demonstrations of EV-mediated metabolic effects toward scalable, clinically relevant strategies that redefine treatment beyond hemodynamics and toward energetic restoration of the failing heart [13]. To contextualize EV-mediated bioenergetic therapy within the current therapeutic landscape, Table 1 compares conventional heart failure treatments with EV-based approaches across mechanisms of action, energetic targets, clinical limitations, and potential synergy.

To contextualize EV-mediated bioenergetic therapy within current heart failure management, Table 1 summarizes the principal mechanisms, energetic effects, limitations, and potential complementarities of established guideline-directed medical therapies. Information was synthesized from contemporary ACC/AHA, ESC, and major heart failure review literature [21,22,23,24,25,26,27,28,29,30,41,42,43,44], whereas EV-related mechanisms were derived from experimental and translational studies focused on mitochondrial and metabolic reprogramming [4,5,13,14,34,35,36].

**Table 1:** Comparative Therapeutic Landscape: EV Bioenergetic Therapy vs. Conventional HF Treatments.

Therapy Class	Primary Mechanism	Energetic Target	Key Clinical Limitation	Synergy Potential with EVs
β-Blockers [22,24,26,27]	Neurohormonal attenuation; heart rate reduction	↓ myocardial oxygen demand (indirect)	No direct restoration of mitochondrial function; energetic deficit persists	EVs may restore ATP production while β-blockers reduce demand
ACEi/ARNI [22,23,24,25]	RAAS modulation; afterload reduction	Secondary metabolic relief via hemodynamic unloading	Energetic inefficiency remains uncorrected	EVs could enhance mitochondrial efficiency during reverse remodeling
SGLT2 Inhibitors [24,27,30,31,43]	Systemic metabolic shift; ketone utilization	Modest improvement in myocardial fuel efficiency	Do not repair mitochondrial structure or ETC dysfunction	EVs may enhance mitochondrial responsiveness to altered substrates
Mineralocorticoid Receptor Antagonists (MRAs) [22,24,25,27]	Aldosterone pathway inhibition; anti-fibrotic and anti-remodeling effects	Indirect preservation of mitochondrial function through reduced fibrosis, oxidative stress, and inflammation	Limited direct restoration of ATP production or mitochondrial biogenesis	EVs may complement MRAs by directly enhancing mitochondrial respiration and bioenergetic efficiency while MRAs reduce adverse remodeling
Diuretics [22,23,24]	Reduction in congestion and ventricular filling pressures	Secondary reduction in myocardial energy demand through improved hemodynamics	Primarily symptomatic benefit; no direct correction of mitochondrial dysfunction	EVs may provide metabolic restoration while diuretics alleviate hemodynamic burden and congestion
Device Therapy (CRT, LVAD) [24,29,44]	Mechanical unloading/resynchronization	Improves mechanical efficiency, not bioenergetics	Intrinsic cellular energetic failure persists	EVs may stabilize myocardial metabolism during unloading
Regenerative Cell Therapy [1,2,4,13,29]	Structural repair; paracrine signaling	Limited, indirect metabolic effects	Variable engraftment; inconsistent energetic impact	EVs provide scalable, non-cellular metabolic coordination
EV Bioenergetic Therapy [4,14,35,36,37,39,44,45]	Intercellular metabolic and mitochondrial reprogramming	ATP generation, redox balance, mitochondrial dynamics	Translational scaling, dosing standardization	Compatible with GDMT, devices, and metabolic modulators

Table 1 provides the comparison of established heart failure therapies and extracellular vesicle (EV)-based bioenergetic interventions across mechanisms of action, energetic targets, clinical limitations, and opportunities for therapeutic synergy. Information regarding guideline-directed medical therapy (β-blockers, ACEi/ARNI, mineralocorticoid receptor antagonists, SGLT2 inhibitors, diuretics, and device-based therapies) was synthesized from contemporary heart failure guidelines and major cardiovascular reviews [21,22,23,24,25,26,27,28,29,30,41,42,43,44]. EV-related mechanisms and proposed bioenergetic synergies were derived from preclinical and translational studies discussed throughout this review [4,5,13,14,34,35,36]. ↓ indicates reduction.

### 1.2. Novelty and Scope of This Review

This review builds upon, but is distinct from, our previous publications on extracellular vesicles (EVs), mitochondrial biology, cardiac regeneration, and heart failure. While several prior articles examined specific aspects of EV biology, mitochondrial transfer, stem-cell-derived vesicles, or regenerative cardiovascular therapies individually, the present work does not reproduce those reviews. Instead, it provides an integrated framework centered on EV-mediated bioenergetic reprogramming as a unifying therapeutic concept in heart failure.

The novelty of this review lies in four major areas. First, it synthesizes evidence across multiple EV cargo classes, mitochondrial quality-control pathways, and cardiac cell populations within a system-level bioenergetic framework rather than focusing on isolated molecular mechanisms. Second, it integrates mitochondrial energetics, metabolic flexibility, redox regulation, and intercellular communication into a single translational model relevant to both HFrEF and HFpEF. Third, it proposes a phenotype-guided clinical positioning strategy for EV therapies, including disease-specific EV sources, delivery approaches, and bioenergetic endpoints. Fourth, it introduces a translational architecture linking mechanistic findings to biomarker development, dosing logic, and integration with contemporary guideline-directed medical therapy.

**Figure 2:**
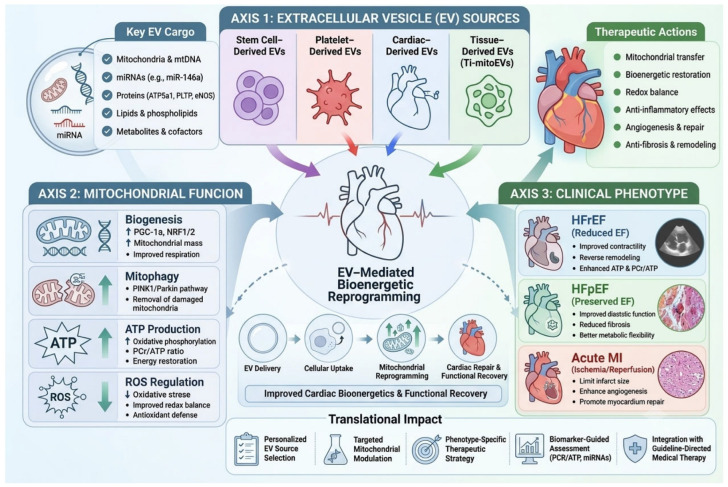
Extracellular Vesicle-Mitochondrial Bioenergetic Axis Across Heart Failure Phenotypes: An Integrated Source–Mechanism–Disease Framework. This flow integrates extracellular vesicle (EV) origin, mitochondrial bioenergetics, and cardiovascular phenotype into a unified axis-based model. 1. EVs are stratified by cellular source—stem cell-derived EVs enriched in regenerative and pro-survival cargo, platelet-derived EVs mediating thrombo-inflammatory and vascular signaling, cardiac-derived EVs reflecting myocardial stress and injury responses, and tissue-derived EVs contributing systemic metabolic–inflammatory cross-talk. 2. These EVs converge on key mitochondrial regulatory nodes, including biogenesis (PGC-1α/NRF1), mitophagy (PINK1/Parkin), ATP production via oxidative phosphorylation, and ROS homeostasis through antioxidant pathways such as Nrf2, with dysregulation leading to progressive energetic failure. 3. The resulting mitochondrial state determines clinical expression across heart failure phenotypes, where HFrEF is dominated by ATP depletion and oxidative stress-driven contractile dysfunction, HFpEF by inflammation-associated energetic inefficiency and impaired relaxation, and AMI by acute mitochondrial injury with ROS surge and ischemia–reperfusion damage. Axis 1: Extracellular vesicle (EV) sources (stem cell-derived EVs/platelet-derived EVs/cardiac-derived EVs/tissue-derived EVs). Axis 2: Mitochondrial function (biogenesis/mitophagy/ATP production/reactive oxygen species regulation). Axis 3: Clinical phenotype (heart failure with reduced ejection fraction [HFrEF]/heart failure with preserved ejection fraction [HFpEF]/acute myocardial infarction [AMI]. ↑ indicates increase, ↓ indicates reduction. Colored arrows from Axis 1 to the center: Indicate that EVs from diverse biological sources (stem cell, platelet, cardiac, tissue) are delivered to and taken up by the heart. Curved arrows from Axis 2 to the center: Represent the specific mitochondrial mechanisms (biogenesis, mitophagy, ATP production, ROS regulation) that drive the “EV-Mediated Bioenergetic Reprogramming” inside cardiac cells. Linear timeline arrows at the bottom center: Show the sequential therapeutic process: EV Delivery → Cellular Uptake → Mitochondrial Reprogramming → Cardiac Repair & Functional Recovery. Large green arrow pointing to “Therapeutic Actions” (top right): Shows that successful bioenergetic reprogramming directly results in systemic healing effects (e.g., mitochondrial transfer, anti-fibrosis, angiogenesis). Large blue arrows pointing to Axis 3: Show how fixing these mitochondrial pathways translates into distinct clinical improvements across specific heart failure phenotypes (HFrEF, HFpEF, and Acute MI). Dashed curved arrows (bottom center to Axis 2 & 3): Represent a feedback loop, illustrating how improved cardiac bioenergetics and functional recovery directly reverse mitochondrial failure and rescue the clinical phenotypes.

To facilitate transparency, the present review should be viewed as a conceptual synthesis rather than an update of any single prior publication by the authors. Previous works examined specific aspects of extracellular vesicle biology, cardiac regeneration, mitochondrial transfer, or cardiovascular therapeutics individually. In contrast, this review integrates these previously separate domains into a unified framework centered on bioenergetic reprogramming in heart failure, supported predominantly by original experimental and translational studies from multiple independent research groups. This concept is elaborated in Table 2 and Figure 2.

## 2. EV-Mediated Bioenergetic Reprogramming in Heart Failure—Mechanisms, Molecular Dynamics, and Translational Scaling

Heart failure (HF) is increasingly understood not merely as a hemodynamic syndrome, but as a system-level energetic disorder marked by profound mitochondrial dysfunction and maladaptive metabolic remodeling, as summarized in the system-level framework shown in Figure 1. In the failing myocardium, mitochondrial oxidative phosphorylation—which underpins ~90% of ATP production—becomes inefficient, with documented reductions in ATP synthesis ranging from 30% to 40%, alongside concomitant decreases in phosphocreatine/ATP ratios that predict adverse outcomes in human HF cohorts [3,5,14].

Healthy adult myocardium derives 60–80% of its ATP from fatty acid oxidation; however, failing hearts demonstrate a metabolic shift toward glycolysis and glucose oxidation [1,2,3,5], accompanied by downregulation of key regulators such as PPARα, PGC-1α, and ERRα. Quantitatively, myocardial ATP content in advanced heart failure is reduced by approximately 30–40% [3,4], while phosphocreatine-to-ATP ratios decline by 20–50% [4,6,7,8]. These energetic deficits reflect impaired electron transport chain (ETC) activity, reduced expression of regulators of mitochondrial biogenesis (e.g., PGC-1α, ERRα), and excess reactive oxygen species (ROS) production, which further uncouples oxidative phosphorylation and damages cellular structures [1,2,3,4,9,10,12]. Collectively, these alterations establish a persistent energy deficit that drives contractile dysfunction, maladaptive hypertrophy, and systemic neurohormonal activation.

Within this landscape of metabolic crisis, extracellular vesicles (EVs) have emerged as endogenous bioenergetic modulators that both reflect and potentially correct failing cardiac metabolism. Unlike classical nanocarrier concepts that frame EVs as passive vehicles for isolated molecular cargo, accumulating evidence indicates that EVs instead orchestrate coordinated bioenergetic reprogramming across cardiomyocytes, endothelial cells, immune cells, and fibroblasts through complex, integrated signaling cargo.

### 2.1. Molecular Mediators of EV-Driven Energetic Reprogramming

EVs encapsulate a rich array of biomolecules capable of influencing energy metabolism at multiple nodes. For example, EVs carrying metabolic enzymes such as ATP synthase subunits (e.g., ATP5a1) can directly augment mitochondrial function in recipient cardiomyocytes [35]. In murine models of ischemic injury, cardiac-derived EVs transfer ATP5a1, suppress mitochondrial ROS, maintain membrane potential, and reduce ferroptosis—outcomes associated with improved contractile performance [35,45,72] (Table 3).

Beyond enzymes, EVs shuttle regulatory RNAs and proteins that modulate mitochondrial dynamics—coordinating fusion/fission processes that are critical for energetic efficiency. EV cargo influences key regulators such as DRP1, MFN1/2, and mitochondrial biogenesis pathways, restoring membrane potential and reducing ROS accumulation in stressed cardiomyocytes.

As summarized in Table 3, EV cargo acts through multiple complementary bioenergetic pathways rather than isolated molecular targets. MicroRNAs primarily regulate transcriptional programs governing mitochondrial biogenesis and oxidative metabolism, whereas transferred enzymes and mitochondrial regulatory proteins support electron transport chain function, TCA-cycle activity, and mitochondrial dynamics. In parallel, redox-active proteins, bioactive lipids, and immunometabolic mediators contribute to maintenance of redox balance, membrane integrity, and intercellular metabolic coordination. Such effects are reproducible across stem cell-derived EV platforms, suggesting conserved mechanisms in metabolic rescue [14].

Importantly, EVs also reprogram substrate utilization. In failing hearts, the relative contribution of oxidative phosphorylation from pyruvate and fatty acids declines, with compensatory upregulation of glycolysis that nonetheless fails to meet ATP demand [5]. EVs carrying modulators of metabolism can influence substrate preference and increase mitochondrial respiration. For example, specific microRNAs (e.g., miR-126, miR-210) delivered by EVs have been shown to enhance oxidative capacity and reduce maladaptive glycolytic reliance, supporting improved ATP generation at the organ level.

### 2.2. System-Wide Integration: Beyond the Cardiomyocyte

While cardiomyocytes are primary targets, the systemic impact of EV-driven bioenergetic reprogramming extends to nonmyocyte populations. In human HF, circulating EVs (CEVs) from patients exhibit a distinct proteomic signature that can provoke inflammatory and lipid metabolic disturbances in naïve cardiomyocytes and fibroblasts, promoting ER stress, autophagy, and pro-fibrotic responses. This underscores that not all EVs are therapeutically beneficial; pathological EVs can aggravate metabolic derangements if their content reflects disease state.

Conversely, engineered or endogenous restorative EV populations can recalibrate immune cell metabolism, encouraging reparative macrophage polarization and reducing pro-inflammatory cytokine cascades linked to metabolic stress. These shifts reduce myocardial oxygen demand and improve tissue energetics—an essential component of global cardiac function.

### 2.3. Quantifying Bioenergetic Effects in Preclinical Models

Preclinical studies provide quantitative benchmarks for EV-mediated energetic improvements. EV treatment in rodent HF models has yielded 20–50% increases in mitochondrial oxygen consumption rates and citrate synthase activity, along with significant reductions in ROS accumulation. Functional outcomes include 5–15% improvements in ejection fraction and up to 20–40% decreases in fibrotic burden compared to untreated controls. These metrics signify not merely molecular correction but organ-level energetic rescue [35,36,37,39,45].

Furthermore, EVs with enhanced mitochondrial content (mitoEVs) are capable of transferring functional mitochondrial components, stimulating mitochondrial biogenesis, and promoting metabolic flux towards oxidative phosphorylation—a critical feature distinct from simple cargo delivery models [36,37,39].

### 2.4. Challenges in Translational Scaling

Despite robust mechanistic data, translating EV-mediated bioenergetic therapies into human HF paradigms faces key challenges. EV heterogeneity—variability in size, cargo composition, and source cell metabolic state—complicates standardization and potency. Additionally, quantitative dosing strategies must be established, involving not only particle counts but functional measures such as ATP-generating capacity, redox modulation, and metabolic reprogramming efficacy.

Immunogenicity and biodistribution also pose constraints, as EVs interact with systemic immune networks that can either enhance or blunt therapeutic effects depending on context. Engineering strategies that tune EV surface markers and metabolic cargo profiles are emerging as solutions to these barriers.

### 2.5. Integrating EV Bioenergetics into Heart Failure Paradigms

**Figure 3:**
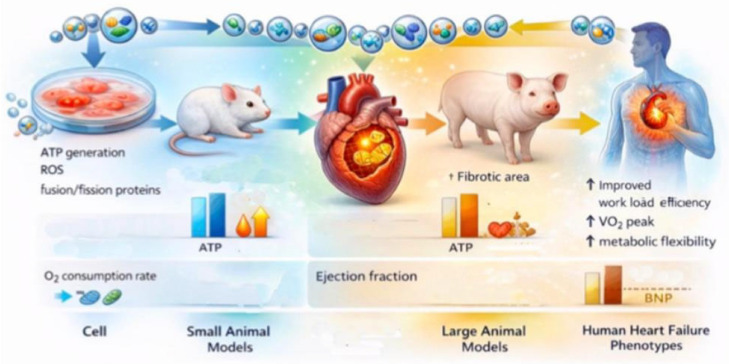
Scaling EV Bioenergetic Effects from Preclinical Models to Heart Failure Phenotypes. This schematic illustrates the progressive scaling of extracellular vesicle (EV)-mediated bioenergetic effects from reductionist preclinical systems to clinically relevant heart failure phenotypes, with the blue-to-gold color gradient representing the transition from in vitro discovery to large-animal and human clinical translation. The top circular icons depict the tracking and delivery of EV components across systems. At the cellular level, EVs enhance mitochondrial respiration, normalize redox balance, and improve ATP efficiency in stressed cardiomyocytes and nonmyocyte populations. These bioenergetic gains translate in small-animal models into measurable improvements in mitochondrial oxygen consumption (20–50%), reductions in oxidative stress, and modest but reproducible increases in cardiac function (e.g., ejection fraction) [3,4]. Validation in large-animal models demonstrates preservation of myocardial energetic reserve, attenuation of fibrotic remodeling (indicated by ↑ fibrotic area text and accompanying tissue quantification graphs), and improved mechanical efficiency under physiological loading conditions. In human heart failure contexts, EV-driven bioenergetic reprogramming is positioned to address shared metabolic deficits across ischemic and non-ischemic etiologies, linking molecular energetic restoration to functional endpoints such as exercise capacity, myocardial oxygen efficiency, and metabolic reserve, correlated with a downregulation of clinical biomarkers like B-type natriuretic peptide (BNP). The left-to-right progression (indicated by block arrows) emphasizes translational continuity, highlighting how integrated bioenergetic mechanisms observed in preclinical models inform scalable therapeutic strategies for clinical heart failure management. For illustration purposes, bar graphs represent relative changes in ATP, ejection fraction, and BNP between control [light bars] and treated [dark bars] groups.

To move toward clinical translation, it is essential to embed EV-mediated bioenergetic reprogramming within existing HF treatment frameworks. This includes combining EV strategies with metabolic modulators (e.g., AMPK activators), hemodynamic therapies, and patient stratification based on metabolic phenotypes (e.g., HFrEF versus HFpEF). The integration of EV therapies could reframe heart failure management not as a problem of contractile support alone, but as system-wide energetic optimization—repairing, enhancing, and sustaining myocardial and vascular metabolic networks [13]. The translational scaling of EV-mediated bioenergetic effects across experimental and clinical heart failure contexts is summarized in Figure 3.

### 2.6. Mitochondrial Quality Control and Mitophagy as Central Targets of EV-Mediated Bioenergetic Rescue

Beyond augmenting ATP production and oxidative phosphorylation, a critical yet underemphasized mechanism of EV-mediated bioenergetic restoration lies in the regulation of mitochondrial quality control (MQC) [35,36]. In heart failure, mitochondrial dysfunction is not solely a consequence of impaired electron transport chain (ETC) activity, but also of defective turnover of damaged mitochondria [19,51]. Accumulation of depolarized mitochondria with disrupted cristae structure leads to excessive reactive oxygen species (ROS) generation, cardiolipin oxidation, and release of pro-apoptotic factors such as cytochrome c [44,52]. These events amplify energetic inefficiency and trigger maladaptive remodeling [44,56].

Mitophagy—the selective autophagic clearance of damaged mitochondria—is a central component of MQC and is tightly regulated by the PINK1–Parkin signaling axis [54,59]. Under physiological conditions, mitochondrial membrane potential (ΔΨm) prevents stabilization of PTEN-induced kinase 1 (PINK1) on the outer mitochondrial membrane [19]. However, in failing cardiomyocytes, depolarization leads to PINK1 accumulation, recruitment of the E3 ubiquitin ligase Parkin, and ubiquitination of mitochondrial surface proteins such as VDAC1 and MFN2, marking them for autophagic degradation [54]. Dysregulation of this pathway in heart failure results in incomplete clearance of dysfunctional mitochondria, perpetuating energetic collapse [19,54].

Emerging evidence suggests that EVs can directly modulate MQC pathways at multiple levels [35,75]. EV cargo containing regulatory microRNAs (e.g., miR-21, miR-210) and proteins has been shown to influence expression of key mitophagy mediators, including PINK1, Parkin, BNIP3, and FUNDC1 [67,75]. For instance, EV-mediated delivery of miR-210 enhances hypoxia-adaptive responses by promoting mitochondrial turnover and optimizing oxygen utilization efficiency [3,75]. Similarly, EV-associated proteins can stabilize mitochondrial membrane potential, indirectly suppressing excessive mitophagy activation while preserving functional mitochondrial networks [36,45].

In addition to regulating degradation pathways, EVs contribute to mitochondrial biogenesis, thereby maintaining mitochondrial population homeostasis [39]. This balance between removal and renewal is orchestrated through transcriptional regulators such as PGC-1α, NRF1, and TFAM [10,35]. EV cargo has been shown to upregulate PGC-1α signaling, leading to increased mitochondrial DNA replication and synthesis of ETC components [10,39]. This dual action—enhancing mitophagy of damaged organelles while promoting biogenesis of functional mitochondria—enables a net improvement in mitochondrial quality rather than simply increasing mitochondrial quantity [35,39].

A further layer of complexity arises from EV-mediated transfer of mitochondrial components themselves [37,46]. While whole-organelle transfer remains debated in terms of efficiency and scalability, EVs enriched with mitochondrial proteins, lipids, and mtDNA fragments can integrate into recipient mitochondrial networks. Cardiolipin, a mitochondria-specific phospholipid critical for ETC supercomplex stability, has been identified within EV cargo and may contribute to restoration of membrane curvature and respiratory efficiency in damaged mitochondria [37,47]. Similarly, transfer of ETC subunits such as ATP5a1 supports reconstitution of ATP synthase activity, directly enhancing bioenergetic output [45,47].

Importantly, MQC modulation by EVs extends beyond cardiomyocytes to other cardiac cell types [32,33]. In fibroblasts, restoration of mitochondrial function reduces activation of pro-fibrotic pathways mediated by TGF-β signaling and metabolic reprogramming toward glycolysis [32,34]. In macrophages, EV-induced shifts toward oxidative metabolism promote anti-inflammatory (M2-like) polarization, reducing cytokine-driven metabolic stress within the myocardium [38,76]. These multicellular effects highlight that mitochondrial quality control is not a cell-autonomous process but a coordinated network phenomenon across cardiac tissue [33,76].

However, the therapeutic manipulation of MQC via EVs presents several challenges [60,61]. Overactivation of mitophagy can lead to excessive mitochondrial depletion, while insufficient activation permits accumulation of dysfunctional organelles [19,54]. Therefore, EV-based interventions must achieve a finely tuned balance, likely requiring context-dependent cargo engineering [14,35]. Additionally, variability in EV composition—driven by donor cell metabolic state, isolation method, and environmental conditions—can influence their impact on MQC pathways, underscoring the need for standardized characterization of mitochondrial regulatory cargo [60,62].

From a translational perspective, MQC-related biomarkers may provide critical readouts of EV therapeutic efficacy [4,77]. These include circulating levels of mitochondrial DNA, expression of PINK1/Parkin pathway components, and imaging-based assessments of mitochondrial turnover [4,5]. Integration of such biomarkers with functional endpoints like ATP production and oxygen consumption will be essential for establishing causal links between EV-mediated MQC modulation and clinical outcomes [8,49].

Collectively, EV-driven regulation of mitochondrial quality control represents a unifying mechanism that integrates bioenergetic restoration, redox balance, and cellular survival pathways [35,59]. By simultaneously enhancing mitochondrial function, removing damaged organelles, and promoting biogenesis, EVs offer a systems-level approach to correcting the energetic deficits that define heart failure [4,14].

## 3. Translational Architecture: Positioning EV Bioenergetic Therapy Across the Heart Failure Spectrum

### 3.1. Disease-Specific Positioning and Clinical Delivery of EV Therapies

While EV-mediated bioenergetic restoration has demonstrated efficacy across multiple experimental models, successful clinical translation will likely require matching EV source and cargo composition to specific heart failure phenotypes. Different EV populations exhibit distinct biological properties, suggesting that a one-size-fits-all approach may not be optimal.

In ischemic heart failure, mesenchymal stromal cell-derived EVs (MSC-EVs) have shown robust effects on mitochondrial preservation, angiogenesis, and attenuation of oxidative stress, making them attractive candidates for post-infarction remodeling and ischemic HFrEF. In contrast, induced pluripotent stem cell-derived EVs (iPSC-EVs) may be particularly suited for non-ischemic cardiomyopathies because of their capacity to enhance mitochondrial biogenesis, metabolic flexibility, and cardiomyocyte-specific signaling pathways. For HFpEF, where endothelial dysfunction, inflammation, and impaired energetic reserve play dominant roles, endothelial-cell-derived or engineered EVs enriched with angiometabolic cargo may provide greater therapeutic benefit by improving vascular–cardiac metabolic coupling.

Consequently, future clinical development may require phenotype-specific EV platforms analogous to contemporary precision medicine approaches in oncology and cardiovascular therapeutics.

### 3.2. Human Delivery Strategies for EV-Based Bioenergetic Therapies

The route of EV administration represents a critical determinant of therapeutic efficacy and clinical feasibility. Intravenous administration remains the most clinically scalable approach because it enables repeated dosing and broad systemic distribution. However, significant EV sequestration by the liver, spleen, and reticuloendothelial system may reduce cardiac delivery efficiency.

Intracoronary delivery provides greater myocardial exposure and has been successfully utilized in multiple cardiovascular cell-therapy studies. This approach may be particularly suitable for ischemic heart failure and post-myocardial infarction remodeling, where targeted delivery to injured myocardial territories is desired.

Direct intramyocardial administration offers the highest local retention and tissue exposure but requires invasive catheter-based or surgical procedures, potentially limiting routine use in chronic heart failure populations.

Emerging bioengineering approaches, including hydrogel-assisted delivery, cardiac-targeting peptides, and surface-engineered EVs, may further enhance myocardial biodistribution while minimizing systemic clearance (Table 4).

**Figure 4:**
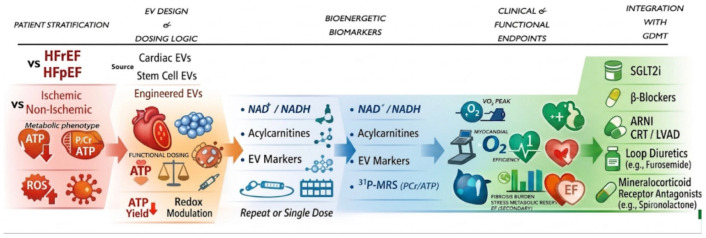
Conceptual framework for the clinical translation of EV-based bioenergetic therapy in heart failure. The schematic illustrates a stepwise pathway from patient stratification based on metabolic phenotype, through EV dosing logic defined by functional bioenergetic potency, to biomarker-guided assessment and energy-centric clinical endpoints. Patient stratification is proposed to identify who benefits energetically, not just clinically. EV design and dosing logic reinforce functional potency over particle number. Bioenergetic biomarkers show energy-centric pharmacodynamics. Marking clinical and functional endpoints helps reframe success beyond EF alone. Integration with GDMT positions EVs as a bioenergetic layer, not a replacement. Integration with guideline-directed medical therapy is emphasized, positioning EVs as a complementary bioenergetic layer capable of enhancing myocardial efficiency and systemic metabolic coordination across heart failure phenotypes. A future translational paradigm may involve EV administration as an adjunct to optimized GDMT, analogous to how contemporary HF management employs layered therapeutic strategies targeting complementary biological mechanisms. Under such a framework, neurohormonal modulation, hemodynamic optimization, and bioenergetic restoration would be viewed as interconnected rather than competing therapeutic domains. Red Arrow (Stratification → Dosing): Represents utilizing the patient’s specific metabolic phenotype (e.g., ATP depletion, ROS levels) to directly inform and customize the design and functional dosing logic of the engineered Extracellular Vesicles (EVs). Orange Arrow (Dosing → Biomarkers): Represents the progression from delivering a targeted EV dose to measuring its real-time, energy-centric pharmacodynamic effects using specific bioenergetic biomarkers (e.g., NAD+/NADH ratios). Blue Arrow (Biomarkers → Endpoints): Represents translating these cellular bioenergetic improvements into measurable, macro-level clinical and functional endpoints (such as improved myocardial O_2_ efficiency and VO_2_ peak on a treadmill). Green Arrow (Endpoints → GDMT): Represents the ultimate integration of EV therapy into standard care, positioning bioenergetic restoration as a complementary, layered strategy that enhances and works alongside established Guideline-Directed Medical Therapy (GDMT).

Figure 4 outlines a proposed clinical translation framework for EV-based bioenergetic therapy, integrating patient stratification, dosing logic, biomarker selection, endpoints, and alignment with guideline-directed medical therapy. A central feature of the proposed framework is phenotype-guided EV selection. Although bioenergetic dysfunction is a shared hallmark across heart failure syndromes, the dominant energetic abnormalities differ substantially between ischemic HFrEF, non-ischemic HFrEF, and HFpEF. Ischemic HFrEF is characterized primarily by loss of viable myocardium, impaired oxidative phosphorylation, and excessive oxidative stress, suggesting potential benefit from EV populations enriched in mitochondrial-protective and angiogenic cargo. In contrast, non-ischemic HFrEF is often associated with diffuse mitochondrial dysfunction and impaired metabolic adaptability, where EVs promoting mitochondrial biogenesis and energetic resilience may be more appropriate. HFpEF exhibits a distinct energetic profile dominated by endothelial dysfunction, inflammation, microvascular impairment, and reduced metabolic flexibility despite relatively preserved myocardial ATP content. Consequently, EVs designed to enhance endothelial-metabolic coupling and immunometabolic regulation may provide greater benefit in this population. These distinctions support a precision-bioenergetic model in which EV composition, cargo profile, and delivery strategy are matched to the predominant energetic deficits of each HF phenotype rather than applied uniformly across all patients.

The clinical translation of EV-mediated bioenergetic reprogramming requires a framework that accounts for the heterogeneity of heart failure phenotypes, disease stage, and metabolic status, consistent with the multi-compartment bioenergetic coordination depicted in Figure 1. Heart failure is not a monolithic entity; ischemic and non-ischemic etiologies, as well as HFrEF and HFpEF subtypes, exhibit distinct metabolic signatures that shape therapeutic responsiveness. For example, HFrEF is characterized by marked reductions in oxidative phosphorylation capacity and mitochondrial density [50,51,52,71,78,79], whereas HFpEF displays preserved ATP levels [53] with impaired metabolic flexibility, endothelial dysfunction, and elevated myocardial stiffness driven by inflammatory and fibrotic energetics [54,55].

EV-based bioenergetic therapies are uniquely positioned to operate across this spectrum because they modulate energy coordination rather than isolated pathways. In ischemic HF, EVs that enhance mitochondrial efficiency, restore NAD^+^/NADH balance, and suppress ROS can directly address energy starvation in viable myocardium [1,3,4,34,35]. In non-ischemic and HFpEF contexts, EV-mediated signaling toward endothelial nitric oxide synthase activation, macrophage metabolic reprogramming, and fibroblast quiescence can reduce myocardial oxygen demand while preserving diastolic function [50,51,52,53,54,55,71,78,79].

Importantly, EV therapy is not envisioned as a replacement for guideline-directed medical therapy (GDMT), but rather as a complementary strategy that may address residual bioenergetic deficits that persist despite contemporary treatment [41]. Although β-blockers, ACE inhibitors/ARNI, mineralocorticoid receptor antagonists, SGLT2 inhibitors, diuretics, and device-based therapies substantially improve survival, reverse remodeling, and clinical outcomes, many patients continue to exhibit impaired mitochondrial function, reduced energetic reserve, and abnormal metabolic flexibility [42,43,44]. These observations suggest that optimization of hemodynamic and neurohormonal pathways does not necessarily equate to complete restoration of myocardial bioenergetic health.

Importantly, several GDMT components exert favorable metabolic effects. SGLT2 inhibitors, for example, promote shifts in substrate utilization, improve myocardial efficiency, and may indirectly influence mitochondrial function, while β-blockers reduce myocardial oxygen demand and mineralocorticoid receptor antagonists attenuate fibrosis-associated metabolic stress [42,43]. Nevertheless, these therapies are not specifically designed to deliver coordinated mitochondrial repair or system-wide bioenergetic reprogramming across multiple cardiac cell populations. In this context, EV-based interventions may provide an additional layer of metabolic regulation by enhancing mitochondrial quality control, redox homeostasis, and intercellular energetic communication. Rather than competing with established therapies, EV-mediated bioenergetic restoration may therefore function synergistically within existing HF treatment frameworks [41,42,43,44].

Consistent with this concept, abnormalities in myocardial phosphocreatine-to-ATP ratios, mitochondrial respiration, and metabolic reserve have been reported even among patients receiving optimized contemporary HF therapy.

Similarly, device-based therapies such as cardiac resynchronization therapy (CRT) and left ventricular assist devices (LVADs) improve mechanical efficiency and ventricular unloading but do not directly target the cellular mechanisms underlying mitochondrial dysfunction. EV-based bioenergetic therapies may therefore complement reverse remodeling by stabilizing myocardial metabolism and supporting restoration of energetic reserve during treatment-induced recovery [44].

From a temporal standpoint, EV bioenergetic therapies may be most effective during early-to-mid stages of HF, when mitochondrial plasticity and metabolic adaptability remain partially intact. However, emerging data suggest benefits even in advanced disease, where EVs can reduce inflammatory metabolic burden and improve peripheral organ energetics, indirectly supporting cardiac performance.

## 4. Defining Therapeutic Efficacy: Bioenergetic Endpoints, Biomarkers, and Dosing Logic

A critical barrier to clinical translation of EV-based therapies is the lack of standardized bioenergetic endpoints [4,35,60,76]. Traditional cardiac endpoints—left ventricular ejection fraction, NYHA class, or natriuretic peptide levels—capture hemodynamic status but incompletely reflect energetic restoration. Because EV therapies target metabolic coordination, efficacy must be assessed using energy-centric metrics.

At the molecular level, candidate biomarkers include circulating NAD^+^/NADH ratios, acylcarnitine profiles, and mitochondrial DNA copy number, which correlate with myocardial metabolic health. Proteomic signatures of circulating EVs themselves may serve as both pharmacodynamic markers and predictors of response. At the tissue level, noninvasive imaging modalities such as phosphorus-31 magnetic resonance spectroscopy (^31P-MRS) allow direct measurement of phosphocreatine-to-ATP ratios [61,64], which decline by up to 50% in advanced HF and improve in response to metabolic therapies [56,65].

Functional bioenergetic outcomes include myocardial oxygen consumption efficiency, exercise capacity (VO_2_ peak) [64], and metabolic reserve under stress rather than resting EF alone [48,57,77,80] (Figure 3). In preclinical models, EV-mediated improvements in mitochondrial respiration of 20–50% translate into modest but meaningful functional gains [34,35,36], suggesting that even partial energetic rescue has clinical relevance.

Dosing paradigms for EV bioenergetic therapies must also evolve beyond particle number [58]. Potency should be defined by functional capacity, including ATP-generating potential, redox modulation, and ability to induce mitochondrial biogenesis [39,47,62] in standardized assays [59]. This represents a conceptual shift similar to that seen in cell therapy, where functional readouts supersede cell counts as indicators of efficacy.

Finally, safety considerations—including immunogenicity, off-target metabolic effects, and long-term energetic reprogramming—must be addressed through rigorous pharmacokinetic and biodistribution studies [63]. Advances in EV engineering, including surface modification and cargo enrichment, offer strategies to enhance cardiac targeting while minimizing systemic exposure.

## 5. Conclusions

Heart failure remains a leading cause of morbidity and mortality worldwide, in large part because current therapeutic paradigms insufficiently address its most fundamental biological constraint: chronic myocardial energetic failure. Across ischemic and non-ischemic etiologies, progressive impairment of mitochondrial efficiency, metabolic flexibility, and intercellular energy coordination limits cardiac performance long before irreversible structural deterioration occurs. This review reframes heart failure not solely as a disorder of pump function or neurohormonal dysregulation, but as a system-level bioenergetic disease amenable to targeted metabolic intervention.

Within this context, extracellular vesicles emerge as a unique and transformative therapeutic platform. Rather than functioning as passive delivery vehicles, EVs act as bioenergetic therapeutics capable of orchestrating coordinated metabolic and mitochondrial reprogramming across cardiomyocytes, endothelial cells, immune populations, and fibroblasts. Preclinical studies consistently demonstrate that EV-mediated signaling restores mitochondrial respiration, reduces oxidative stress, and improves energetic efficiency at the organ level, translating into measurable functional gains even in chronically remodeled myocardium. Importantly, these effects scale beyond isolated molecular pathways, reflecting a systems biology mechanism aligned with the multifactorial nature of heart failure.

This review integrates mechanistic, preclinical, and translational evidence to address a critical question in cardiovascular therapeutics: how EV-driven bioenergetic reprogramming can be deployed within real-world heart failure treatment paradigms. By situating EV therapies alongside guideline-directed medical therapy and device-based interventions, we propose a complementary strategy that enhances energetic resilience rather than replacing existing standards of care. Such integration is particularly relevant across the heart failure spectrum, including HF with reduced and preserved ejection fraction, where metabolic dysregulation remains a shared yet under-targeted pathology.

Looking forward, successful clinical translation will require standardized EV manufacturing, functional potency metrics rooted in bioenergetic performance, and energy-centric biomarkers capable of capturing therapeutic impact beyond hemodynamic endpoints. As advances in EV engineering, metabolic phenotyping, and noninvasive energetic imaging converge, EV-based bioenergetic therapies have the potential to redefine heart failure management—from symptomatic support toward systemic metabolic renewal.

By advancing a system-level framework that bridges preclinical insights to translational application, this work positions EV-mediated bioenergetic reprogramming as a foundational pillar in the next generation of heart failure therapeutics.

## Figures and Tables

**Table 2:** Novel Conceptual Contributions of This Review and Supporting Literature.

Novelty Domain	Specific Contribution of This Review	Evidence
1. System-level integration of EVs, mitochondria, and cardiac regeneration	Proposes EV-mediated bioenergetic reprogramming as a unified framework linking mitochondrial dysfunction, intercellular signaling, and cardiac repair rather than isolated mechanisms	EV/mitochondria: [36,37,39,45,46,47]; mitochondrial HF foundation: [19,44,48]
2. Integrated mitochondrial energetics + metabolic flexibility model across HF phenotypes (HFrEF + HFpEF)	Synthesizes ATP-PCr depletion, ROS dysregulation, mitophagy impairment, and substrate shifts into a single HF metabolic continuum	[7,8,28,41,49,50,51,52,53,54,55,56,57,58,59]
3. Phenotype-guided translational positioning of EV therapy integrated with GDMT	Proposes disease-stage-specific EV sources, delivery strategies, and combination with guideline-directed medical therapy (GDMT)	HF therapeutics: [22,23,24,25,26,30,31,43]; metabolic therapy integration: [27,28,57]
4. Translational architecture linking mechanism → biomarker → therapy design → clinical implementation	Introduces a translational pipeline: mechanistic EV cargo → mitochondrial function readouts → biomarker development (PCr/ATP, EV cargo) → dosing/standardization frameworks	EV translation/standardization: [60,61,62,63]; 31P-MRS energetics biomarkers: [7,49,64]; systems modeling: [65]
5. Advanced engineering layer (iPSC, CRISPR, biomaterials, EV engineering convergence)	Integrates CRISPR-guided mitochondrial engineering, biomaterial maturation platforms, and EV-based delivery systems for next-generation cardiac regeneration	[1,2,3,10,12,14,35,36]
6. Multi-modal regulatory and intercellular signaling expansion (miRNA, endothelial EV axis, redox systems)	Expands EV framework to include miRNA-mediated metabolic control and endothelial-mitochondrial cross-talk in cardiac disease	[59,66,67,68,69,70,71]

While prior publications from the authors contributed to specific components of EV biology, mitochondrial metabolism, and regenerative strategies, those works are cited selectively for continuity and do not constitute the conceptual synthesis presented in this review.

**Table 3:** EV Cargo and Bioenergetic Targets Across Cardiac Cell Types.

EV Cargo Class	Representative Cargo	Primary Target Pathways	Bioenergetic Outcomes	Supporting Models
miRNAs [34,35,36]	miR-126, miR-210, miR-21	PGC-1α signaling, mitochondrial biogenesis, redox balance	↑ oxidative phosphorylation, ↓ maladaptive glycolysis, ↑ ATP efficiency	Murine HF, ischemic cardiomyocytes, stem cell–EV platforms
Metabolic enzymes [35,37,38,39,40]	ATP5a1, citrate synthase	ETC complex stabilization, TCA cycle flux	↑ mitochondrial respiration, ↑ ATP production, ↓ ROS	Rodent ischemia/reperfusion, HF models
Mitochondrial regulatory proteins [1,3]	MFN2, OPA1 modulators	Fusion–fission balance, cristae integrity	Restored mitochondrial dynamics, improved Ca^2+^ handling	In vitro cardiomyocyte stress models
Redox regulators [3,4]	Antioxidant enzymes, NAD^+^-modulating proteins	NAD^+^/NADH homeostasis, ROS detoxification	↓ oxidative stress, improved coupling efficiency	HF plasma EV exposure studies
Bioactive lipids [1,2,73]	Cardiolipin-associated lipids, sphingolipids	Membrane fluidity, ETC supercomplex stability	↑ O_2_ utilization efficiency, ↓ proton leak	Cardiac and endothelial EV studies
Inflammation-modulating cargo [34,35,36,66,67,74]	miR-146a, immunometabolic proteins	Macrophage polarization, cytokine signaling	↓ inflammatory metabolic burden, ↓ myocardial O_2_ demand	Murine HF, immune–cardiac co-culture
Angiometabolic factors [68,69,70]	eNOS-associated regulators	Endothelial–cardiomyocyte metabolic coupling	↑ perfusion efficiency, ↑ substrate delivery	Vascular EV studies

Table 3 summarizes the principal classes of extracellular vesicle (EV) cargo involved in myocardial bioenergetic regulation, including microRNAs, metabolic enzymes, mitochondrial regulatory proteins, redox-active molecules, bioactive lipids, and immunometabolic mediators. These cargo components influence mitochondrial function, substrate utilization, redox homeostasis, and intercellular metabolic communication, collectively contributing to improved energetic efficiency and metabolic resilience in preclinical heart failure models. ↑ indicates increase, ↓ indicates reduction.

**Table 4:** Proposed Clinical Positioning of EV Therapies in Heart Failure.

HF Subtype	Candidate EV Source	Main Bioenergetic Mechanism	Potential Delivery Route
Ischemic HFrEF	MSC-EVs	mitochondrial rescue, angiogenesis	intracoronary, IV
Non-ischemic HFrEF	iPSC-derived EVs	mitochondrial biogenesis	IV
HFpEF	endothelial EVs, engineered EVs	endothelial-metabolic coupling	IV
Advanced HF/LVAD	engineered EVs	systemic energetic support	IV

Table 4 summarizes the candidate EV sources, mechanisms, and delivery routes based on currently available preclinical and early translational evidence and intended to illustrate a conceptual framework for phenotype-guided clinical development rather than established clinical recommendations. Intravenous (IV) administration offers scalability and repeat dosing potential, whereas intracoronary delivery may enhance myocardial exposure in ischemic settings. Further clinical studies are required to determine optimal EV source selection, dosing strategies, biodistribution, and route-specific efficacy.

## Data Availability

No additional unpublished datasets or statistical code were generated.
